# Keeping an eye on circadian time in clinical research and medicine

**DOI:** 10.1002/ctm2.1131

**Published:** 2022-12-25

**Authors:** Elizabeth B. Klerman, Allison Brager, Mary A. Carskadon, Christopher M. Depner, Russell Foster, Namni Goel, Mary Harrington, Paul M. Holloway, Melissa P. Knauert, Monique K. LeBourgeois, Jonathan Lipton, Martha Merrow, Sara Montagnese, Mingming Ning, David Ray, Frank A. J. L. Scheer, Steven A. Shea, Debra J. Skene, Claudia Spies, Bart Staels, Marie‐Pierre St‐Onge, Steffen Tiedt, Phyllis C. Zee, Helen J. Burgess

**Affiliations:** ^1^ Department of Neurology Massachusetts General Hospital, Brigham and Women's Hospital Boston Massachusetts USA; ^2^ Division of Sleep Medicine Harvard Medical School Boston Massachusetts USA; ^3^ Plans Analysis, and Futures John F. Kennedy Special Warfare Center and School Fort Bragg North Carolina USA; ^4^ Alpert Medical School of Brown University Department of Psychiatry and Human Behavior EP Bradley Hospital Chronobiology and Sleep Research Providence Rhode Island USA; ^5^ Department of Health and Kinesiology University of Utah Salt Lake City Utah USA; ^6^ Sir Jules Thorn Sleep and Circadian Neuroscience Institute Nuffield Department of Clinical Neurosciences University of Oxford Oxford UK; ^7^ Biological Rhythms Research Laboratory Department of Psychiatry and Behavioral Sciences Rush University Medical Center Chicago Illinois USA; ^8^ Neuroscience Program Smith College Northampton Massachusetts USA; ^9^ Radcliffe Department of Medicine University of Oxford Oxford UK; ^10^ Section of Pulmonary Critical Care, and Sleep Medicine Department of Internal Medicine Yale School of Medicine New Haven Connecticut USA; ^11^ Sleep and Development Laboratory Department of Integrative Physiology University of Colorado Boulder Boulder Colorado USA; ^12^ Boston Children's Hospital and Kirby Neurobiology Center Boston Massachusetts USA; ^13^ Institute of Medical Psychology Faculty of Medicine LMU Munich Germany; ^14^ Department of Medicine University of Padova Padova Italy; ^15^ Clinical Proteomics Research Center and Cardio‐Neurology Division Massachusetts General Hospital Harvard Medical School Boston Massachusetts USA; ^16^ NIHR Oxford Biomedical Research Centre John Radcliffe Hospital Oxford UK; ^17^ Oxford Centre for Diabetes Endocrinology and Metabolism University of Oxford Oxford UK; ^18^ Medical Chronobiology Program Division of Sleep and Circadian Disorders Departments of Medicine and Neurology Brigham and Women's Hospital Boston Massachusetts USA; ^19^ Oregon Institute of Occupational Health Sciences Oregon Health and Science University Portland Oregon USA; ^20^ Chronobiology Faculty of Health and Medical Sciences University of Surrey Guildford UK; ^21^ Department of Anesthesiology and Intensive Care Medicine Charité – Universitaetsmedizin Berlin Berlin Germany; ^22^ Univ Lille Inserm CHU Lille Institut Pasteur de Lille U1011‐EGID Lille France; ^23^ Division of General Medicine and Center of Excellence for Sleep and Circadian Research Department of Medicine Columbia University Irving Medical Center New York New York USA; ^24^ Institute for Stroke and Dementia Research University Hospital LMU Munich Germany; ^25^ Center for Circadian and Sleep Medicine Division of Sleep Medicine Northwestern University Feinberg School of Medicine Chicago Illinois USA; ^26^ Sleep and Circadian Research Laboratory Department of Psychiatry University of Michigan Ann Arbor Michigan USA

**Keywords:** chronobiology, chronomedicine, circadian, circadian medicine, daily, diurnal, human, time‐of‐day, translational

## Abstract

**Background:**

Daily rhythms are observed in humans and almost all other organisms. Most of these observed rhythms reflect both underlying endogenous circadian rhythms and evoked responses from behaviours such as sleep/wake, eating/fasting, rest/activity, posture changes and exercise. For many research and clinical purposes, it is important to understand the contribution of the endogenous circadian component to these observed rhythms.

**Content:**

The goal of this manuscript is to provide guidance on best practices in measuring metrics of endogenous circadian rhythms in humans and promote the inclusion of circadian rhythms assessments in studies of health and disease. Circadian rhythms affect all aspects of physiology. By specifying minimal experimental conditions for studies, we aim to improve the quality, reliability and interpretability of research into circadian and daily (i.e., time‐of‐day) rhythms and facilitate the interpretation of clinical and translational findings within the context of human circadian rhythms.

We describe protocols, variables and analyses commonly used for studying human daily rhythms, including how to assess the relative contributions of the endogenous circadian system and other daily patterns in behaviours or the environment. We conclude with recommendations for protocols, variables, analyses, definitions and examples of circadian terminology.

**Conclusion:**

Although circadian rhythms and daily effects on health outcomes can be challenging to distinguish in practice, this distinction may be important in many clinical settings. Identifying and targeting the appropriate underlying (patho)physiology is a medical goal. This review provides methods for identifying circadian effects to aid in the interpretation of published work and the inclusion of circadian factors in clinical research and practice.

## Graphical abstract

Understanding the contribution of the endogenous circadian system to observed time‐of‐day rhythms can yield insights that further health‐related research and optimize clinical treatments and increase life expectancy. This manuscript provides guidance on best practices in measuring and distinguishing endogenous circadian rhythms from time‐of‐day rhythms in a variety of settings.

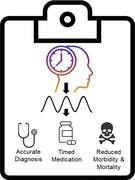

## 1 BACKGROUND

**FIGURE 1:**
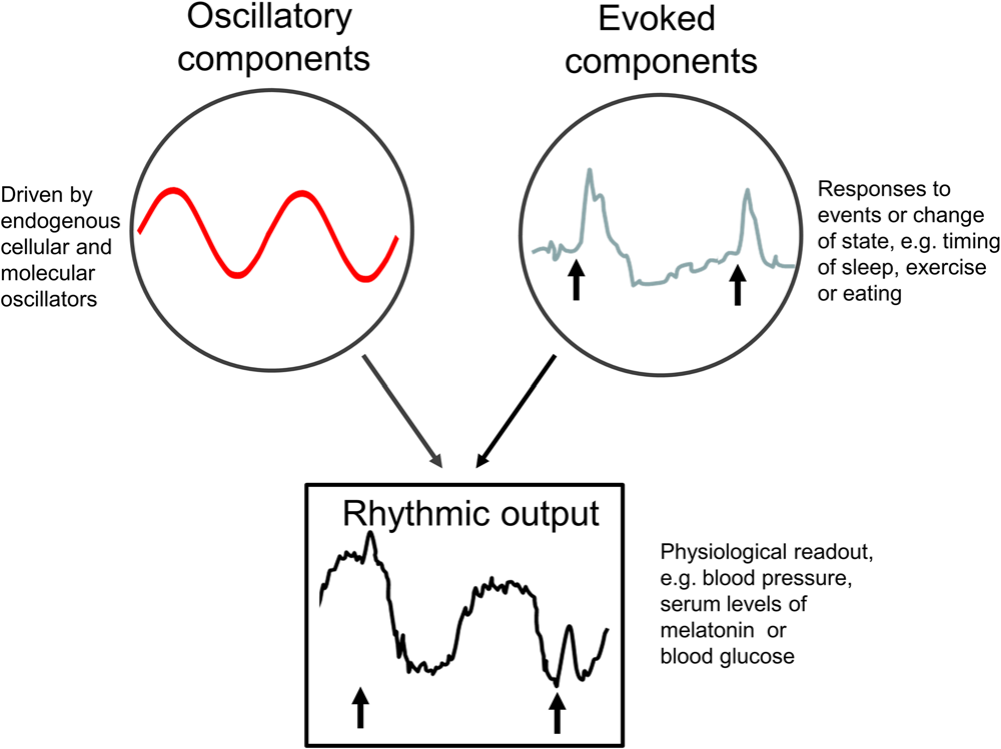
Oscillatory and evoked components contribute to rhythmic outputs. Daily rhythms in physiology (such as blood pressure, glucose, hormone levels, and the rates of distribution, metabolism and excretion of substances) are a composite of both internal oscillatory components driven by cellular and molecular clocks and evoked components in response to external stimuli or behavioural change. Understanding these relative contributions allows for them to be targeted by circadian medicine. Adapted from Klerman and Czeisler *Recent Progress in Hormone Research* 1999.

Human physiology demonstrates multiple rhythms including annual (e.g., seasonal), monthly (e.g., menstrual), daily (diurnal or circadian), ∼90‐min (e.g., NREM‐REM sleep cycles), ∼1 s (e.g., cardiac) and msec (e.g., neuronal). In this paper, we focus on determining the endogenous circadian (i.e., endogenously generated biological rhythms of approximately 24‐h periodicity) component of observed daily (i.e., time‐of‐day) rhythms that result from combined circadian and behaviourally‐ or state‐evoked components (Figure 1). Almost every cellular, physiological and behavioural process has an endogenous circadian rhythm component. Human circadian rhythms are generated in both central oscillators (i.e., within the suprachiasmatic nuclei [SCN] of the hypothalamus) and peripheral oscillators (e.g., in liver, heart, kidney, and other tissues). Under normal conditions, these central and peripheral rhythms are synchronized and prepare the body for the anticipated daily behavioural and environmental changes. The circadian system confers physiological advantages under normal conditions but may be counterproductive when behaviours or daily environmental cycles are misaligned with the internal circadian clock—as can occur with shift work, jet lag and some disease states including severe mental illness, neurodevelopmental disorders and diabetes.^1^, ^2^, ^3^, ^4^, ^5^, ^6^ The importance of circadian rhythms in the maintenance of health and the diagnosis, treatment and monitoring of individuals with different disorders^3^, ^5^, ^7^ is gaining recognition. Disease states or disorders, in addition, can disrupt circadian rhythms (e.g., mood disorders can affect circadian timing, neurogenerative disorders can affect circadian amplitude), and people's behavioural responses to illness (e.g., dark home environment, irregular sleep‐wake cycles) can also feedback to disrupt circadian rhythms.

**FIGURE 2:**
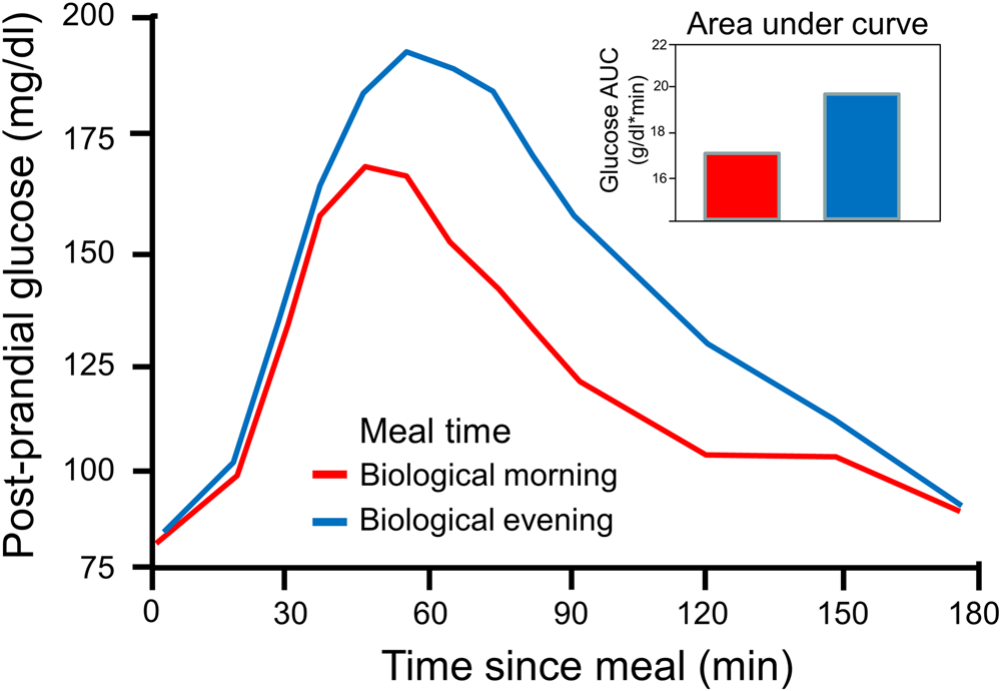
Meal timing relative to the circadian clock influences blood glucose levels. Postprandial glucose profiles differ depending on whether a nutritionally identical meal is consumed in the biological morning (red) or biological evening (blue), with impacts not only on the temporal profile of blood glucose levels but also on the cumulative total blood glucose (area under curve shown in inset). This highlights the importance of circadian timing on human health and disease management (such as diabetes). Adapted from Morris, C. J., et al., *Proceedings of the National Academy of Sciences* 2015.

The field of human chrono‐*physiology* has relied on highly controlled studies of mostly very healthy adult populations and a few clinical populations (e.g., asthma, epilepsy, Parkinson's disease^6^, ^8^, ^9^). Such studies provide clinically relevant information. One example is that the glucose response to meals depends on circadian phase (i.e., timing) of eating (Figure 2); this has implications for metabolism, and may impact the management of disease such as obesity and diabetes.^1^, ^10^ Using chrono‐*pathophysiology* approaches in circadian medicine has been advocated,^11^ but the toolkit for its routine application is not ready. We consider two aims for chrono‐pathophysiology related to circadian medicine: (1) document the role and functioning of the circadian system in disease (with implications for circadian‐based interventions); and (2) assess, diagnose, monitor and/or treat disease‐related circadian disruption or disorders. Clinically relevant examples include increased response/decreased side effects when medications (e.g., for asthma, cancer, hypertension^12^, ^13^) are given at specific clock or circadian times. For both aims, accurate assessment of metrics of human circadian rhythms is required. We recognize that implementing circadian medicine will require knowledge of endogenous circadian and behaviourally/environmentally influenced daily rhythms and their interactions. For example, is the worsening of asthma that typically occurs at night, due solely to the endogenous circadian rhythm, or due to the behavioural/environmental influences typical of night time (e.g., lying down, sleeping, exposure to dust‐mites in mattress), or to a combination of interacting circadian, behavioural and environmental influences?^6^

Therefore, it is important to understand the contribution of the endogenous circadian component to observed rhythms (Figures 1 and 2); this includes understanding the contributions of both endogenous circadian rhythms and daily behavioural and environmental factors to these observed rhythms (Section 2.1.1) so that both these factors (circadian and daily) can be targeted by circadian medicine. For example, high postprandial glucose levels could be due to disrupted circadian rhythms (e.g., from shiftwork) or to eating at an inappropriate circadian time; the two causes would require different interventions. Another important distinction is that circadian disruption is more than disruption of sleep patterns: circadian disruption can independently worsen physiology above and beyond sleep disruption.^6^, ^14^ Both sleep/wake per se and circadian rhythms have independent and interacting effects on many physiological processes, can impair the other, and can have negative impacts on health and disease.

We aim to provide guidance on best practices in measuring metrics of endogenous circadian rhythms for use by clinicians and researchers who are not circadian specialists as they evaluate publications or plan their own experiments of circadian or daily rhythms in humans. Specifying the minimal experimental conditions for such studies should improve the quality and reliability of the work and support and promote the inclusion of circadian rhythms assessments in studies of health and disease. Secondarily, we wish to facilitate the interpretation of clinical and translational findings within the context of human circadian function. Due to current limitations in the assessment of peripheral circadian oscillators, this review will focus on assessment of the central circadian clock, with occasional consideration of measurements of peripheral clocks.

We begin with a review of environmental and physiological variables that affect circadian rhythms (and therefore must be considered when designing experiments) and relevant animal studies that inform human circadian biology. We continue with human physiology (examples in Section 2.2.1) and pathophysiology (reviewed in^3^). We then describe the different protocols, variables, and analyses commonly used for studying human daily rhythms; this includes how to assess the relative contributions of the endogenous circadian system and other daily patterns in behaviours or the environment. We also include definitions and examples of circadian terminology (Table 1) and guidelines for collecting protocol variables (Table 2).

**TABLE 1:** Terminology: Definitions and examples

Term (Abbreviation)	Definition	Notes and examples
Actogram	A graphical representation of a variable over time, such that cycles are plotted vertically.	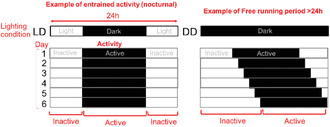
Amplitude	Amplitude is half of the difference between peak and trough (i.e., the difference between the peak [or trough] and the mean value).	Note: A full cycle of data is required to establish an amplitude. 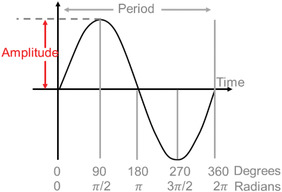
Biological day/night	The time of day or night as defined by the circadian system, even if it differs from the environmental time/condition.	Note: also ‘circadian day/night’ and ‘subjective day/night’.
Chronobiology	The study of biological rhythms, both endogenously and exogenously driven.	Multiple time frames are possible: For example, molecular, ultradian (<24), circadian (∼24 h), lunar/tidal, annual.
Chronotype	Individual timing of behaviours (e.g., activity and rest) within the 24‐h day. We assume that chronotype reflects the entrained phase of the individual's circadian clock (see Entrainment below).	Chronotype is dependent on an individual's genes, age, sex, timing of light exposure and social factors (e.g., work/school/family schedules). Note: May be either a preference (e.g., from the Horne‐Östberg questionnaire) versus timing of event (e.g., from the Munich Chronotype Questionnaire).
Circadian day/night	The time of day or night as defined by the circadian system, even if it differs from the environmental time/condition.	Note: Also ‘biological day/night’ and ‘subjective day/night’ Example(s): (1) in constant lighting (including dark) conditions in nocturnal animals, the subjective day is their habitually inactive circadian phase(s); (2) in jet lag or shift work, an individual may be awake and working during their subjective night.
Circadian Desynchrony	An uncoupling of two rhythms; only one must be a circadian oscillator. ‐ External. Steady state in which a biological rhythm runs with a different period than an external zeitgeber. ‐ Internal. Steady state in which different biological rhythms within one organism, or different components of the same biological rhythm run with different periods.	Note: Compare with circadian misalignment (i.e., phase) in which the two rhythms can have the same period.
Circadian Rhythm	A biological rhythm with a period of approximately 24 h. Note that for a biological rhythm to be classified as ‘circadian’, it (1) must be endogenously generated from a self‐sustained oscillator; (2) can be synchronized to an environmental cycle (e.g., by the light/dark cycle); and (3) must be temperature compensated (i.e., maintain robust rhythms with an ∼24‐h periodicity over a range of physiological temperatures).	Note: In human studies, to determine whether rhythms are driven by the endogenous circadian system, and not a secondary ‘masking’ consequence of behaviours, such as sleep, activity and food intake (often driven by non‐circadian mechanisms, especially in humans), specific protocols are required that separate the timing of circadian rhythms from behaviours, or carefully control behaviours as in a constant routine protocol.
Circadian System	A network of circadian oscillators that can coordinate/interact such that they are appropriately aligned. Note: A testable hypothesis is that this is an optimal temporal structure for physiology.	Note: This may include both central (e.g., in the suprachiasmatic nuclei [SCN] in mammals) and peripheral oscillators.
Circadian Misalignment	Abnormal timing (phase) between different cycles, at least one of which is circadian. A circadian rhythm in abnormal phase (timing) relationship(s) with other cycles (e.g., with the environmental light/ dark cycle or other circadian rhythms).	Notes: (1) Subtypes (not mutually exclusive): Environmental circadian misalignment Behavioural circadian misalignment Internal circadian misalignment (2) Compare with desynchrony in which the two rhythms have different periods.
Circadian time (CT)	Time scale covering one full circadian period. Zero point is defined arbitrarily. Note: This is usually assessed in constant dark in nocturnal rodents. For those conditions, CT12 is usually onset of locomotor activity.	Note: Compare with zeitgeber time (ZT) below.
Daily/diel/ rhythm Day/night rhythm	A rhythm that recurs on a daily basis in the real world.	Note: These rhythms may not be endogenous circadian rhythms. Therefore, we suggest the use of ‘daily’ or ‘time‐of‐day’ instead of ‘circadian’ in such cases.
Diurnal	Formally, this refers to a rhythmic behaviour that peaks or is more likely to occur during the light phase or daytime.	Note: Compare with nocturnal.
Entrainment	Coupling of a self‐sustained oscillator to a zeitgeber (the forcing oscillation) with the result that either both oscillations have the same frequency (synchronization) or that the frequencies are integral multiples (frequency de‐multiplication) (e.g., 12‐ and 24‐h periodicities).	Note: This is possible only within a limited range of frequencies.
Free‐running period (FRP):	The length of time it takes for an organism's endogenous rhythm to complete one cycle in the absence of a zeitgeber/environmental time cue.	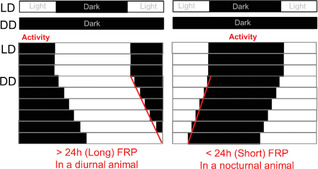
Light metrics	Favored metrics: Radiometric measures refer to electromagnetic energy within the optical spectrum, which includes ultraviolet radiation, visible light and infrared radiation. Irradiance is a measure of radiometric energy from all directions over a 180° field of view. Radiance is a measure of radiometric energy viewed from a specific direction or region in space.^105^ Less favored metrics: Photometric measures refer to human visual responses to visible light that falls between the wavelengths of 400–700 nm. A photopic response (maximum sensitivity at a wavelength of 555 nm), and a scotopic response (maximum sensitivity at a wavelength of 507 nm). Illuminance (illumination) is a photometric measure of light from all directions over a 180° field of view. Luminance is a photometric measure of light measured or viewed from a specific direction or region in space.	Note: Details in Methods in Enzymology paper.^105^ Irradiance units include μW/cm^2^ and photons/cm^2^/s. Radiance units include μW/cm^2^/sr and photons/cm^2^/s/sr. Illuminance metrics include lux (lx) and lumen (lm)/m2. Luminance include lumen (lm)/m2/sr. Note: Photometric units (lux) are not recommended because they measure brightness as it would appear to the human visual ‐ not the circadian ‐ system. Note: Since rods, cones and the melanopsin‐based systems all contribute to the circadian response, melanopic metrics alone may not reflect all these inputs.
Masking	A driven biological process that obscures or overrides an underlying circadian rhythm. Masking stimuli include but are not limited to light, activity, food and sleep.	Examples: Activity in mice suppressed when lights are turned on, blind people's activity is influenced by local clock time 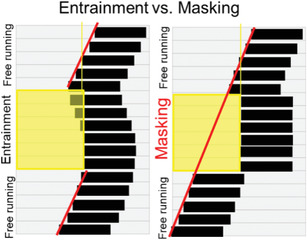 .
Nocturnal	Formally, this refers to a rhythmic behaviour that peaks or is more likely to occur during the dark phase or night‐time.	Note: Compare with diurnal.
Period	The length of time it takes to complete one cycle. This may be an endogenous period (τ, ‘tau’) or a forced period (Τ, in which biological processes [e.g., sleep/wake schedules] or zeitgebers are used).	Sometimes referred to as τ (‘tau’). Note: The Τ‐cycle may not entrain the process (e.g., in a forced desynchrony protocol), which may result in masking. 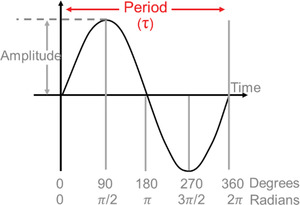
Phase(φ)	Any defined point on an oscillation (e.g., peak, trough, onset or offset). Reflects a time point on the rhythm.	Note: Usual symbol is (φ) 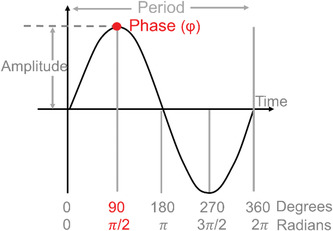
Phase angle	The difference between two phases. Typically, a zeitgeber cycle and a biological oscillation or two stable biological oscillations (e.g., beginning of activity onset relative to lights on or beginning of sleep relative to onset of melatonin synthesis).	Note: This should be used instead of the term ‘phase angle difference’. 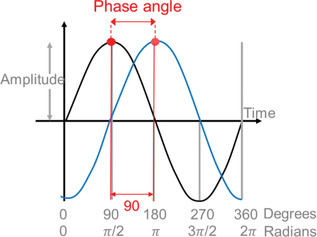
Phase response curve (PRC)	A plot defining the relationship between the timing of a zeitgeber exposure (e.g., light) and the change in phase in response to that stimulus at that time.	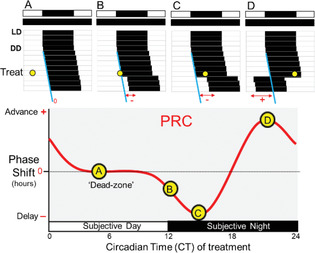
Phase shift (Δφ)	A change in the phase of a circadian cycle induced by zeitgeber exposure.	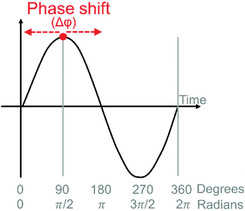
Sleep episode	A time window from sleep onset (e.g., from polysomnographically defined sleep) to the last epoch of sleep within a bedrest episode. It may include intervening sequences of wakefulness.	Notes: (1) This is preferred over ‘sleep period’. (2) This is different from sleep ‘opportunity’ or ‘bedrest episode’.
Social jet lag (SJL)	The phenomenon whereby socially dictated sleep/wake time (e.g., by an alarm clock) differs from circadian clock regulated sleep/wake time.	Notes: (1) measured in hours (2) typically, social jet lag is associated with sleep loss. 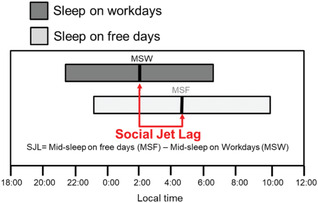
Subjective day/subjective night.	The time of day or night as defined by the circadian system, even if it differs from the environmental time/condition.	Note: See also referred to as ‘circadian day/circadian night’ or ‘biological day/biological night.’ Example: The subjective night of an evening chronotype person may be 2:00 AM to 11:00 AM.
Tau [τ]	Greek symbol refers to the period of biological rhythm.	Note: See Period.
Zeitgeber	A stimulus that can shift the phase of the circadian clock.	Note: (1) when these stimuli are presented rhythmically, the circadian system is entrained to them. Examples: Light, food, serum shock.
Zeitgeber time (ZT)	Time scale covering one full zeitgeber cycle.	Note: ZT0 is typically defined as the time lights turn on.

**TABLE 2:** Variables recommended to be collected with gold standard, compromise and pragmatic/proxy measurement techniques

Variable	Gold standard	Compromise	Pragmatic/Proxy markers of daily (not circadian) rhythms
Light	Continuous wearable sensor attached near the face. Monitors intensity and spectrum of light	Continuous wearable sensor on wrist or lapel with monitoring of intensity	Single reading at the angle of gaze
Circadian phase	Dim light melatonin onset (DLMO)	Other blood, urine, or saliva‐based biomarkers	Behavioural rest/activity rhythms
Sleep	Polysomnography	Wrist‐worn actigraphy, preferably with additional sensors (e.g., heart rate, temperature, respiration).	Wrist‐worn actigraphy, sleep diaries
Chronotype	Questionnaire	Actigraphy of sleep midpoint	
Food intake	Energy and macronutrient intakes at eating occasion (including those that exclusively contain beverages) over multiple days.	Total energy and macronutrient intakes across each hour or waking versus sleeping times of a 24‐h day.	

## 2 DESIGNING, CONDUCTING AND ANALYZING HUMAN CIRCADIAN RHYTHMS STUDIES

When evaluating publications claiming circadian rhythmicity in a variable or outcome, there are two major challenges. The first is that many publications report results as ‘circadian rhythms’, when in fact they are daily rhythms. Daily rhythms are typically measured in conditions with ongoing daily environmental and behavioural changes (e.g., light/dark and sleep/wake cycles). Thus, these daily changes reflect both endogenous circadian rhythms plus evoked effects from behaviours or states (whose timing is influenced by endogenous circadian rhythms), such as postural changes, eating, sleeping and physical activity or environmental inputs (e.g., light, temperature, noise) (Figure 1). While physiological and behavioural changes across the day are important, assuming that they are *solely* driven by the circadian system confounds efforts to understand and/or target underlying circadian physiology. A second challenge is that many published experiments have not been conducted in well‐controlled conditions. Two examples are: (1) experiments with uncontrolled light levels during sample collections for melatonin; this will confound the assessment of circadian effects on melatonin since nocturnal melatonin synthesis is suppressed by room lighting, and (2) experiments that are scheduled based on time‐of‐day rather than adjusting for a participant's habitual sleep/wake timing. Since sleep/wake timing is an imperfect marker of circadian phase (Section 2.3.6), aligning protocol schedules to habitual sleep/wake timing reduces the variance across individuals in circadian phases of any measurements and reduces variance associated with participants shifting their sleep/wake timing and associated behaviours prior to study.

Although guidelines and recommendations exist for designing circadian experiments and collecting or analysing data,^15^, ^16^, ^17^, ^18^ none address all these components, as we attempt here. Standardized protocols and analyses for human studies of all types, including population and clinical research are needed for the discipline to progress. We focus on field (e.g., out‐of‐laboratory) studies, since highly controlled, prolonged in‐laboratory protocols that are the hallmark of many circadian studies are not always feasible due to the demands on participants, requirements for facilities, personnel, equipment and expense. Other issues in planning experiments are related to specific populations: (1) patients may show more variability in data metrics than healthy controls,^19^ potentially due to disease processes or daily variation in disease‐related behaviours such as the timing of medication, (2) patients might exhibit extremes in phenotype/behaviour not accounted for in reference populations and (3) the relationship of the physiology/behaviour may be different in patients than in healthy controls. Therefore, assumptions derived from studies of healthy controls about relationships (e.g., relative timing of circadian phase to sleep/wake cycles) may not be valid in those populations.

### 2.1 Daily rhythmicity in humans

Human physiology is highly rhythmic on a daily scale due to both endogenous circadian rhythms and responses to the environment (e.g., light/dark) and behaviours (e.g., sleep, eating, physical activity).^20^ The mammalian circadian system is hierarchically organized such that the central pacemaker in the SCN of the hypothalamus orchestrates timing and amplitude of circadian rhythms of multiple physiological functions and of the peripheral oscillators. The SCN is composed of a network of coupled neurons and glia that express a near‐24‐h oscillation of gene expression and neuronal activity.^21^, ^22^ The SCN receives light input via the retino‐hypothalamic tract and uses these light signals to synchronize with the external environment.^23^ SCN‐based timing information is relayed by both neuroanatomical projections within and beyond the hypothalamus,^24^ by release of poorly understood humoral timing cues into cerebrospinal fluid, and via control of melatonin secretion. Since the SCN tightly controls the timing of melatonin secretion, measures of melatonin are considered gold standard for estimating central endogenous circadian phase (Section 2.3.2). The timing cues impacting the *peripheral* oscillators are highly tissue specific (e.g., food timing affecting gut, liver and pancreatic clocks^25^).

#### 2.1.1 Variables that influence the expression of circadian rhythms

Rhythms are synchronized to a cycle by zeitgebers (i.e., stimuli that can shift the clock's phase) (Table 1). Zeitgebers may affect central and peripheral tissue circadian clocks differentially (e.g., SCN vs. liver) and even affect cells differentially within a tissue.^26^ In addition, there are probably feedback and feedforward loops between zeitgebers and the circadian system; for example, light exposure shifts the phase of circadian rhythms, which may then alter the timing of subsequent light exposure.

##### Light stimuli

Organisms use the 24‐h light/dark cycle to entrain their endogenous circadian rhythms to an appropriate phase.^27^ In mammals, light stimuli for circadian rhythms are processed only through the eyes. The time (phase), intensity, spectral composition, duration, history and pattern^27^ of the light stimulus affect circadian phase and amplitude. An individual's response to light also depends on many inter‐individual differences, including age, chronotype and genotype (see below).

##### Non‐photic (i.e., non‐light) stimuli

Non‐photic zeitgebers, including locomotor activity and food intake,^28^ have been identified in animals, but the strength of these non‐photic cues in humans is less clear and less well‐studied.^28^ No non‐photic zeitgebers have been found that are as strong as light. Clocks in peripheral organs are synchronized by the central circadian pacemaker in the SCN, rhythms of body temperature and hormones, and the timing of external behaviours, including food intake.^25^

Exogenous melatonin or its analogues administered at an appropriate circadian time can entrain the endogenous circadian rhythms of people who are totally blind^29^, ^30^ and shift circadian rhythms in sighted people.^31^ Appropriately timed physical activity^32^ and caffeine^33^ can shift the timing of the SCN‐driven melatonin rhythm and presumably the endogenous circadian system but do not entrain the circadian system in totally blind people.^34^ Caffeine may alter the circadian system's response to light.^33^, ^35^, ^36^

##### Other variables that affect circadian rhythms

###### Chronotype

Chronotype refers to a preference for or actual behavioural activity timing. There exists a range of times for preferred or actual wake and activity and for self‐reported better mood and cognitive function. For morning‐types, these times are early in the day while for evening‐types these times are later in the day; most people are not at either extreme. Chronotype is influenced by age, sex and genetic variation across many genes,^37^ some of which associate with a longer or shorter intrinsic circadian period.^38^ Chronotype affects alignment with the external environment, and the phase of alignment between internal oscillators. Chronotype is assessed using (1) self‐reported preference for specific times of activities (e.g., Horne‐Östberg questionnaire, also called the Morningness‐Eveningness Questionnaire) [MEQ]^39^; (2) the time of sleep and wake on free days (assumed to reflect circadian timing) and work/school days (assumed to reflect social constraints) by the Munich Chronotype Questionnaire (MCTQ)^40^ including the 5‐question μMCTQ^41^; or (3) inferred as a result of wearable technology capturing behavioural rest‐activity or other rhythmicity. Social Jet Lag (SJL), which is related to chronotype, is calculated as the difference between timing of mid‐sleep on free versus work/school days; it has been correlated with multiple negative health outcomes.^42^ Chronotype correlates with the dim light melatonin onset (DLMO, Section 2.3.2),^43^ which is the current gold standard for assessing circadian phase. Even a single question from the MCTQ asking people to self‐select their chronotype has been clinically useful in predicting future disease course.^44^

The parent‐report Children's Chronotype Questionnaire provides three measures of circadian preference in 2‐ to 11‐year‐olds. These include midsleep time on free days and a multi‐item morningness/eveningness score, both of which correlate with DLMO.^45^

###### Age

There are multiple changes in circadian rhythms with age, including: chronotype^40^ (delayed phase of the circadian system from early childhood to late adolescence, and advanced phase with aging), decreased amplitude of some markers of the circadian system^46^, ^47^ and reduced light sensitivity.^48^, ^49^, ^50^, ^51^ One reason for these changes may be altered anatomy with aging (e.g., lens transparency or pupil diameter).^47^, ^52^

###### Biological sex

Women's chronotype becomes later than men's during adolescence; then men have later chronotype than women until around the age 50.^40^ Women have a shorter period of the human pacemaker^53^ and an earlier timing of circadian phase relative to sleep/wake timing (i.e., larger phase angle [Table 1]).^54^

###### Disease states

It is hypothesized that some disease states may affect circadian rhythm phase and amplitude directly (i.e., not via zeitgebers). Documentation of disease states that may do this (see examples in Section 1) is an active area of investigation.

#### 2.1.2 Translating pre‐clinical work

While this review is focused on methods for studying circadian rhythms in humans, relevant pre‐clinical work in animals is usually considered when planning such studies. Key differences between humans and other mammals must be considered for translation from pre‐clinical models to humans. The most obvious is that most studies use nocturnal rodent species. Despite the difference in behavioural patterns between nocturnal and diurnal mammals, the physiology and function of the SCN are remarkably similar across the 24‐h day with peak SCN neural activity during the habitual light phase and maximal light‐induced resetting during the habitual dark phase in *both* diurnal and nocturnal species^55^; therefore, the nocturnal versus diurnal timing of behavioural rhythms are determined downstream of the SCN.^55^ Experiments performed during habitual human working hours, therefore occur during a rodent's inactive phase and a human's active phase. The differences in the relative relationships of circadian timing and behavioural rhythms between diurnal and nocturnal species may represent a significant confounding factor .^56^, ^57^

The photosensitive retinal ganglion cells (pRGCs; also known as melanopsin retinal ganglion cells or intrinsically photosensitive retinal ganglion cells [ipRGCs]) provide the primary conduit for light information reaching the SCN; this circuit is also modulated by rod and cone inputs within the retina.^23^ Another important species difference is that photoreceptors differ markedly in their spectral responses between mice and humans.^23^ This may have implications for translation of studies of the effects of light from rodents to humans.

A third consideration is that multiple mouse strains do not produce melatonin.^58^ This may affect the physiology under study.

### 2.2 Protocols

Multiple experimental protocols have been used to describe and quantify circadian metrics in humans. These range from highly controlled experiments, to field/home or hospital‐based, to epidemiological studies. Highly controlled studies can be used to collect data from a relatively small group of individuals in whom the effects of almost all potential confounders are minimized. These studies can also be used to identify factors that must be considered for studies that are not as highly controlled. Standardized protocols for studies conducted in non‐research environments (e.g., at home or in hospital) are needed because some people can not or will not be studied in highly controlled environments and because important information can only be collected in those other environments.

#### 2.2.1 Highly controlled protocols

The first highly controlled experiments of human circadian rhythms were conducted in bunkers or caves.^59^, ^60^ These experiments were conducted in environments with relatively constant environmental conditions (e.g., temperature, light) and participants had no access to time‐of‐day information. Non‐24‐h schedules were imposed, or participants were allowed to self‐select their schedules. Since there is a circadian influence on sleep timing, allowing individuals to choose their schedules resulted in specific behaviours (e.g., sleep, eating) occurring only at a limited set of circadian times; therefore, these protocols could not fully separate circadian from time‐of‐day effects. These experiments, nonetheless, demonstrated the importance of circadian rhythms to human physiology.

Since then, two classic protocols have been developed to isolate circadian system effects from the effects of behavioural and environmental influences (Section 2.1.1).^61^ These protocols require multiple days in a laboratory under constant dim‐light conditions (ideally <5 lux^62^) to minimize light effects on the circadian clock. One protocol is the ‘constant routine’ (CR), in which environmental, behavioural and postural changes are minimized for at least 24 h,^63^ and the other protocol is the ‘forced desynchrony’ (FD)^64^ in which all behaviours, including sleep/wake, are scheduled evenly across the entire circadian cycle by imposing identical recurring behavioural cycles with non‐24‐h artificial ‘day’ lengths. Some potential participants are not willing or able to enroll in CR or FD protocols.

For both of these protocols, an individual's behaviour before entering the laboratory portion of the study is usually controlled at home, including regular sleep episodes of at least 8 h/day for at least 1 week to ensure that the individual has stable circadian timing and is not sleep deprived. These regular sleep episodes are also used to schedule the timing of events in‐laboratory. In addition, caffeine, tobacco, alcohol and other substances that are known to affect sleep/wake are usually not allowed for at least 1 week.

Once the participant enters the laboratory, conditions typically include (1) a diet without caffeine and standardized meals (approximately constant in nutrients, calories, and fluids) provided at regular intervals and (2) highly regulated behaviours (e.g., timing of eating and sleeping, amount of activity) for multiple days. In the CR protocol, environmental, behavioural and postural changes are minimized for at least 24 h.^63^ To accomplish this, participants are asked to stay awake in a semi‐recumbent posture in constant dim light and constant room temperature. Circadian rhythms in many variables have been observed in CR protocols, including circulating melatonin, cortisol,^20^ catecholamines,^65^ leptin, glucose and insulin^66^; core body temperature (CBT)^67^; pulmonary function^68^; blood pressure^65^; mood^69^; and cognitive performance.^70^ It is noted that the accumulating sleep loss may affect the variables measured.^71^ The effects of the circadian system and the accumulating sleep loss interact in nonlinear ways; separation of these effects is difficult to achieve. The findings of physiological rhythms assessed with a CR protocol are usually very similar in magnitude and phase compared to the longer FD protocol that better minimizes sleep loss.

In a FD protocol, participants live on non‐24‐h cycles (‘T‐cycles’) of sleep, wake and associated behaviours (e.g., eating, posture changes, social interactions) and environmental changes (e.g., lighting) with cycle periods sufficiently different from 24‐h such that internal clocks cannot synchronize to the imposed T‐cycle; frequently used T‐cycles are 90 min and 28 h.^64^ As a result, the behaviours and endogenous circadian system become desynchronized. Food timing in these protocols does not appear to affect central circadian rhythms,^72^ but there may be effects on peripheral oscillators or the interaction between central and peripheral oscillators,^73^ similar to shift work. By standardizing and distributing all behaviours evenly across the circadian cycle, the FD protocol permits statistical determination of: (1) an average circadian rhythm in a variable while controlling for behavioural effects (by aligning data to each individual's circadian timing); (2) an average effect of the imposed and standardized behavioural cycle on a variable while controlling for any underlying circadian effects (by aligning data to the T‐cycle's period); and (3) any linear or non‐linear interaction between these effects. This allows quantifying if the variable's levels (e.g., responses to a behaviour such as sleep initiation, eating, or response to a medication/intervention) are different at different circadian times.

#### 2.2.2 Controlled measurements in the hospital/clinic or field

Studies that are not conducted using the highly controlled protocols of Section 2.2.1 must still control (or document) highly influential zeitgebers (and other factors) if circadian metrics are to be accurately assessed. Key zeitgebers and other factors include light exposure, sleep schedules, meal timing, ambient temperature, posture and exercise timing and intensity (Table 2, Sections 2.2.1 and 2.5). Adjustments in the home or hospital environment (e.g., lighting levels) for collection of circadian data are possible but require a detailed understanding of the environment and tracking of the intended environmental changes (e.g., lowering light in hospital rooms without impeding clinical care). In addition, other factors must be documented, including intake of circadian‐ or sleep‐influencing substances such as caffeine, nicotine, marijuana, recreational drugs and prescription and non‐prescription medications. Drugs that affect the probability of sleep will also affect the light/dark exposure associated with sleep/wake state and thereby (indirectly) affect circadian rhythmicity.

For young children (2–5 years of age), studies to measure circadian rhythms are best performed in the home.^74^ This increases family compliance and reduces arousal levels in children, and children are more likely to fully participate.

Studies evaluating metabolism must control and document what is eaten, including energy and dietary macronutrient composition, and the timing of food intake. Field‐based tools for the assessment of diet and timing include single‐questionnaire items, 24‐h recalls, food diaries and photograph‐based smartphone apps, adapted to integrate timing information.^75^ The Automated Self‐Administered 24‐h (‘ASA‐24’) recall developed by the National Cancer Institute now includes questions related to timing of sleep episodes and should be used to capture information on dietary intakes and sleep timing in free‐living situations.^76^

#### 2.2.3 Epidemiologic studies

The same issues that arise in Section 2.2.2 also apply to epidemiological studies aimed at estimating circadian metrics (Section 2.3), although the control of relevant variables will likely be more difficult. Most studies will provide time‐of‐day, rather than circadian rhythms data. The simple addition of habitual sleep/wake timing questions would allow for (imperfect) estimation of circadian phase (Section 2.3.6).

#### 2.2.4 Re‐analyses of previously collected data

The same issues that arise in Section 2.2.2 also apply for re‐analyses of already collected data (e.g., from patient electronic health records or from studies in which circadian metrics were not the original variables of interest).

### 2.3 Measurement and analyses for calculating circadian metrics

There are three relevant metrics of endogenous circadian rhythms: period, amplitude and phase (timing). Circadian period is not expected to be relevant for clinical uses except in specialized cases (e.g., circadian rhythm sleep‐wake disorders) or studies of basic human physiology and therefore are not discussed here. Below, we discuss measurement and analysis of circadian phase and amplitude using biomarkers. The non‐linear interactions of circadian and evoked factors (Section 2.2.1) mean that mathematical separation of the two is difficult; there are no validated analysis techniques for this for data not collected under FD conditions.

#### 2.3.1 General considerations for collection of circadian metrics

Criteria for use of a biomarker as a circadian metric should meet the following requirements: (1) it is robust and has shown proof‐of‐concept in highly controlled in‐laboratory settings; and (2) it can be relatively easily measured (e.g., using sensor technologies or 1 or 2 collections of biological samples). Recent development and refinement of sweat biosensors^77^ and microdialysis of hormones and metabolites within interstitial fluids^78^ should allow multiple observations per day (or per hour) in field conditions.

#### 2.3.2 Melatonin

The current gold standard biomarker of phase and amplitude of the central circadian clock is melatonin measured in saliva, blood, or urine, even though it requires more than two sample collections. A key fact relevant to the use of melatonin as a circadian rhythm biomarker is that melatonin production by the pineal gland is predominantly controlled by the SCN^24^, ^79^: the timing of the onset of the melatonin rhythm is tightly associated with other physiological changes including changes in CBT, sleepiness and EEG activity^80^ and reflects the start of the biological night as regulated by the SCN.

Melatonin levels are typically very low during the day but begin to rise 1–3 h before habitual sleep onset time, remain elevated during the night and return to low daytime levels usually within 1 h of habitual wake time.^81^

Melatonin concentrations can be affected by factors that interfere with assessment of the endogenous rhythm: (1) melatonin is suppressed by light even at levels lower than typical room levels,^62^, ^82^ (2) postural changes and exercise can increase melatonin levels,^83^ and (3) substances such as non‐steroidal anti‐inflammatory drugs (NSAIDs), beta‐blockers and alcohol suppress melatonin.^84^, ^85^, ^86^ When studying clinical populations taking medications that affect melatonin, melatonin levels can be assessed, but the potential confounding effect of such medications should be acknowledged.

A washout period without NSAIDs (typically 72 h) and caffeine and alcohol (24 h) should occur prior to sample collection for melatonin. During collection, individuals remain seated and do not eat or drink for at least 10 min prior to each sample collection and do not engage in exercise during the sample collection period.^83^ Sampling occurs every 30 min in dim light (<5 lux) beginning at least 6–7 h before habitual bedtime and continuing until habitual bedtime or later. The primary melatonin metabolite in urine, 6‐sulfatoxymelatonin, can also be assessed, although precision in estimating circadian timing is lost since it is often only feasible to collect urine every 2–4 h, which is partially overcome by multiple days/nights of urine collection.

Measuring melatonin in clinical settings such as hospitals or nursing homes can occur somewhat more easily than in other field environments since there is familiarity with biohazard sample collection and patients are typically less mobile, which facilitates controlling for or documenting posture and mobility and light exposure. Lighting, however, can be difficult to control in these institutional settings. Home salivary melatonin assessments have been used in research and clinically; they depend on participant reliability and require the participant to remain in dim light (often challenging in the home environment), with a fixed posture briefly before data collection, accurately monitoring sample time and number, and storing and shipping the samples appropriately.^87^

**FIGURE 3:**
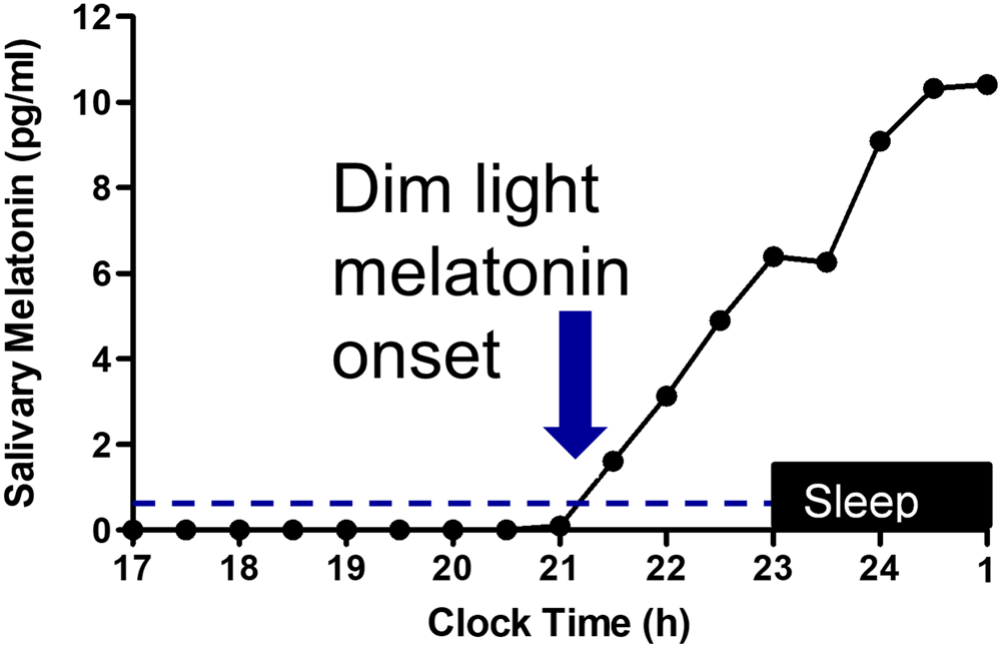
Melatonin levels are low during the daytime and begin to rise 1–3 h before habitual sleep onset. The dim light melatonin onset (DLMO) occurs when the endogenous melatonin levels rise above a low threshold such as 3 pg/ml for saliva or 2 standard deviations above the low baseline points.

Currently the gold standard circadian phase marker in humans is the DLMO.^88^, ^89^ While melatonin levels in saliva are only about 30% of those observed in blood, the timing of DLMO in both sources is similar. The amplitude of the melatonin rhythm can also be estimated with curve fitting methods if the entire melatonin rhythm is sampled overnight^90^; this is not an issue for some blood collection methods but requires the participant to be awake for night‐time saliva sampling. There are multiple melatonin assays that are commercially available, but only some are accurate at the low levels of melatonin required for accurate determination of the DLMO.^91^ For saliva sampling, it is recommended that an absolute or relative threshold be used in the calculation of the DLMO, such as 3 pg/ml or 2 standard deviations above the low daytime baseline points (Figure 3).^17^, ^92^ Note that 10 pg/ml is the most common DLMO threshold for plasma melatonin in adults.

#### 2.3.3 Temperature

CBT can only be used to calculate circadian phase and amplitude under CR or FD conditions due to heavy influences (‘masking’) by multiple behaviours.^93^

#### 2.3.4 Cortisol

Cortisol and other endocrine outputs are unreliable for estimating endogenous circadian phase unless measured under very tightly controlled circadian protocols, such as the CR or FD: (1) cortisol has both circadian and ultradian (i.e., periods significantly less than 24 h) components, and therefore blood sampling at least every 30 min is recommended, (2) sleep itself suppresses cortisol levels^20^ and (3) cortisol production is highly responsive to physiological arousal.

#### 2.3.5 Other physiological markers

Ambulatory metrics such as blood pressure, heart rate, blood glucose or skin temperature have two major limitations: (1) their signals are masked by sleep‐wake state, activity levels, fasting or eating, and postural effects and thus measure daily rather than circadian rhythms; and (2) they currently rely on wearable devices with varying degrees of invasiveness, patient tolerance, reliability and consistency across measurement devices. Some of these metrics (e.g., blood glucose) may eventually be used to measure timing of peripheral clocks.

#### 2.3.6 Behavioural markers

Behavioural markers such as rest/activity timing are not accurate reflections of endogenous circadian timing; obvious examples are shift work and people who are blind. The timing relationship between rest‐activity patterns and circadian timing also depends on the age^94^ and sex^54^ of the individual and whether they have a sleep disorder.^95^ The relationship can vary widely (by 4–5 h) even in healthy, young, and unmedicated individuals maintaining the same sleep‐wake cycle.^94^

#### 2.3.7 Omics‐based technologies (e.g., proteomics, metabolomics, transcriptomics)

Untargeted and targeted omics technologies have been used to develop biomarkers of the central and peripheral circadian clocks.^96^ An advantage of these approaches is that only one or two samples are required to determine circadian phase. Discovery is the first step of omics approach; careful validation and targeted investigation coupled with meticulous clinical phenotyping and innovative bioinformatic analysis are required to overcome prior challenges.^97^ Most omics‐derived biomarkers remain to be validated in clinical populations and in different conditions, such as shiftwork.

#### 2.3.8 Recommendations if validated circadian phase markers are not available

As noted above, there are multiple conditions in which the metrics listed below do not reflect the timing of the central SCN circadian pacemaker. Therefore, if validated circadian markers are not available, the results should be described as ‘time‐of‐day’ or ‘daily’ effects, not ‘circadian’.

##### Questionnaires

Chronotype questionnaires (Section 2.1.1) can be used; most are not validated in populations that are not healthy and not routinely sleeping at night.

##### Sleep timing

Sleep timing can be assessed with questionnaires, daily diaries and wearables. The exact wording of questions may affect results.^98^ For most wearables, the performance of the algorithms for estimating time‐in‐bed without user input is generally not validated. The performance of current wearable technology for estimating time‐in‐bed versus actual sleep timing likely varies across populations and the device used.

Online (i.e., internet‐based) sleep timing data collection is preferred over paper‐based data collection. Online allows for data entry to be time‐stamped, allows immediate error checking (e.g., inappropriate date, am/pm errors) by participants, allows staff to perform close‐to‐real time online error checking and checking of whether participants are entering data, reduces recall bias, allows timely feedback from staff to participants, eliminates legibility issues, eliminates staff data entry (with checking for entry data errors) and eliminates loss of paper forms.

### 2.4 General statistical considerations

There are multiple considerations in deciding appropriate analyses, including statistics, of data from human circadian rhythm protocols.^99^ These considerations include: (1) Multiple observations from an individual are usually correlated and therefore require specific statistical methods; longitudinal ‘mixed methods’ analyses are appropriate and more powerful than repeated *t*‐tests that assume the different time points are independent. (2) The number of participants in each group; many studies have relatively few participants because of the expense of the protocol or a limited number of people in the relevant population, meaning that the underlying distribution of the data may not be known. (3) The distribution of data; circadian rhythm, sleep and activity data may not be statistically normally distributed. The underlying distribution of the data will determine: (a) if transformations should be performed before analyses, and (b) appropriate summary statistics. For example, actigraphy counts are not statistically normally distributed and therefore means and standard deviation summary statistics may not be accurate reflections of the underlying data; a more appropriate distribution may be a zero‐inflated Poisson.

The study team should include a statistician when designing the study, analysing the data, in determining criteria for excluding any ‘outliers’, and handling missing data.

Errors in data analyses can occur because of the ‘circular’ nature of time. Attention to times around midnight is essential. For example, the difference between 23:00 and 2:00 is 3 h, not 21 h. Sleep episodes may start on one day and finish the next day. Care in the data input and coding of analysis is required so that appropriate statistics (e.g., sleep duration or mid‐sleep times) are calculated.

### 2.5 Facilitating circadian studies

#### 2.5.1 Currently available measures (Table 2)

The first step is to consider circadian rhythms as an important variable in the study. Circadian rhythms can be an exposure, moderator, mediator or outcome^100^; their specific role will affect the data collection and analysis plans.

We strongly discourage developing new unvalidated questionnaires or changing/freely translating the wording of questions on validated questionnaires, since slight differences^98^ may affect interpretation by the user and will limit applicability, reproducibility, and meta‐analyses, and the new questionnaire will need to be validated. The use of existing questionnaires in new populations may require re‐validation.

For the design, execution and analyses of studies of circadian phase and amplitude as either input or outcome measures, the following guidelines are presented. All should be included as potential covariates in analyses.

##### Document time of every event and sample collection

A source may be written, electronic medical records and/or hospital policy documents. This encompasses timing of collection of biological samples and treatment administration, meal timing, lights on/off times, and administration of hypnotics or other drugs. Notes should be made of whether the event timing is scheduled versus actual time of event; whenever possible, the latter should be used.

##### Document chronotype and SJL

Chronotype and SJL assessment are discussed in Section 2.1.1.

##### Collect information about sleep timing

Use standardized questionnaires to collect data about habitual sleep timing. For adults, this includes the MCTQ, the Pittsburgh Sleep Quality Index^101^ and others in the NIH PhenX Toolkit^®^. For children, this includes the Children's Sleep‐Wake Scale^102^ and Children's Sleep Habits Questionnaire.^103^

##### Collect information about shift work and recent travel across time zones

The duration of time (e.g., months/years) working shift work (morning, evening, night or rotating) should be documented along with the shift hours, direction of rotation and number of shifts per week. If shift work is no longer pursued, then note the date of the last shift.

The number of time zones crossed, and the date(s) and direction of crossing should be documented. The date of the study relative to any transitions between standard time and daylight saving time should be noted.

For some studies, delaying the start of data collection until at least 4 weeks after shift work, 1 week per time zone crossed, and 1 week after transition between standard time and daylight saving time should be considered.

##### Collect information about immediate prior sleep timing and duration

Details of sleep timing assessment are discussed in Section 2.3.6 and Table 2.

##### Collect information about habits

These habits include times spent indoor/outdoor; timing, amount and content of food eaten; and timing of exercise.

Document the time and amounts of substances taken that may affect sleep or wake, including caffeine, alcohol, tobacco and prescription, recreational or over‐the‐counter medications.

##### Collect information about light exposure

Tracking light exposure, the most influential zeitgeber, is particularly important and can be hard to accomplish accurately.^104^ For in‐laboratory or hospital/clinic studies, the light source with spectra and intensity can be identified, although intra‐room variability may still occur and the direction of angle of gaze of the participant will affect light exposure. The light sensing device needs to be located where accurate light exposure to the eye can be approximated (e.g., as a necklace); in cold climates during the winter, wrist worn devices may be covered with clothing and will not accurately measure light exposure.

##### Collect information about medical conditions and medications that may affect circadian rhythms and sleep

Collect information about all prescription and over‐the‐counter medications and dietary supplements; as noted above, some drugs affect melatonin concentrations and sleep propensity.

##### Design studies and analyses with circadian and sleep variables in mind

Work with a statistician in designing the study and analysing the data. Consider the number and relative timing of samples needed for accurately determining any rhythm's phase or amplitude. Timing should consider both clock time and time relative to awakening.

#### 2.5.2 Methods, techniques and resources to be developed

Circadian phase and amplitude markers of central and peripheral circadian clocks are required that are easy to obtain (e.g., one or two biological samples, continuous minimally‐invasive monitoring). All of these markers, including the current ‘omics’ markers, must be validated in different, well‐phenotyped populations and in different conditions (e.g., jet lag, shiftwork, presence of caffeine [in food, beverage, other sources] or other substances). An advantage would be if these markers were available in close‐to‐real time. Such markers may need to be combined with other data (e.g., mathematical models of prediction of circadian phase based on age and light exposure) to increase accuracy.

Publicly available, well documented, and validated analysis tools will enable researchers without analysis expertise to analyse their data and for uniformity of analysis across investigators. Appropriate educational materials will be required so that these tools are used correctly, including how to handle missing data.

Data harmonization efforts (e.g., standards for data collection and documentation) are key for later data storage and use, especially for meta‐analyses and re‐analyses of data.

Storage of data on publicly available open access websites (e.g., The National Sleep Research Resource; https://sleepdata.org/) with appropriate metadata documentation is important for the ability to analyse already collected data.

Working with implementation scientists is important to incorporate knowledge gained into clinical practice so that the goals of improving health are realized.

## 3 CONCLUSION

Both circadian rhythms and daily (i.e., time‐of‐day) effects are important in research and clinical practice, even if they are difficult to distinguish in participants’ habitual living conditions. Two approaches are useful for the further development of the field of medicine: (1) gathering information on ‘daily’ variations allows assessment of their combined impact on treatment outcomes and can be hypothesis generating. (2) Including more detailed circadian rhythm metrics in study design, conduct and analyses allows documenting their importance so that appropriate targeted interventions in health and disease can be designed. Therefore, collection of even limited information (Section 2.5.1) is encouraged. This review provides methods for identifying circadian effects for research or clinical practice so that the examination and eventual inclusion of circadian factors in clinical research and practice can be achieved.

## CONFLICT OF INTEREST

Klerman ‐ Consulting: American Academy of Sleep Medicine foundation, Circadian Therapeutics, National Sleep Foundation, Sleep Research Society Foundation. Yale University Press. Partner owns Chronsulting. Brager ‐ Consulting: National Academy of Sports Medicine, O2X Max Human Performance, Voices in Sport, WHOOP, Inc., Momentous, Inc., Molecule, Inc., LifeAid Beverage Company, Inc. Carskadon ‐ Editor in Chief, Sleep Research Society gold open access journal SLEEP Advances. Depner ‐ Travel support from Sleep Research Society, Consulting: Elsevier Inc. Foster ‐ Circadian Therapeutics, National Sleep Foundation. Goel ‐ NG serves on the Board of Directors of the Sleep Research Society, as President‐Elect (June 2021–June 2022) and as President (June 2022–Present). She receives a stipend for these officer roles. NG also serves on the Board of Directors of the Sleep Research Society Foundtion and on the Board of Directors of the Associated Professional Sleep Societies. NG received an honorarium for a research presentation at Northwestern University Grand Rounds. Harrington ‐ None. Holloway ‐ None. Lipton ‐ None. Knauert ‐ Member of Serca LLC, American College of Chest Physicians. LeBourgeois ‐ None. Merrow ‐ None. Montagnese ‐ None. Ning‐ None. Ray ‐ None. Scheer ‐ served on the Board of Directors for the Sleep Research Society and has received consulting fees from the University of Alabama at Birmingham; his interests were reviewed and managed by Brigham and Women's Hospital and Partners HealthCare in accordance with their conflict of interest policies and his consultancies are not related to the current work. Shea ‐ None. Skene ‐ None. Spies ‐ Personal fees from Georg Thieme Verlag. Dr. Spies has patent 10 2014 215 211.9 licensed, 10 2018 114 364.8 licensed, 10 2018 110 275.5 licensed, 50 2015 010 534.8 licensed, 50 2015 010 347.7 licensed and 10 2014 215 212.7 licensed. Staels ‐ None. St‐Onge‐ None. Tiedt ‐ None. Zee ‐ Consulting: Eisai, Jazz, Atria Health, CVS Caremark, Idorsia, stock ownership: Teva. Burgess ‐ Consulting: Natrol, LLC, Moving Mindz, Pty Ltd, F. Hoffmann‐La Roche Ltd.
